# Association between inflammation, lipopolysaccharide binding protein, and gut microbiota composition in a New Hampshire Bhutanese refugee population with a high burden of type 2 diabetes

**DOI:** 10.3389/fnut.2022.1059163

**Published:** 2023-01-06

**Authors:** Brandy Moser, Dustin Moore, Bishnu Khadka, Carrie Lyons, Tom Foxall, Cheryl P. Andam, Cooper J. Parker, Chinedu Ochin, Mahdi Garelnabi, Joseph Sevigny, W. Kelley Thomas, Sherman Bigornia, Maria Carlota Dao

**Affiliations:** ^1^Department of Agriculture, Nutrition, and Food Systems, University of New Hampshire, Durham, NH, United States; ^2^Building Community in New Hampshire, Manchester, NH, United States; ^3^Department of Biological Sciences, University of New Hampshire, Durham, NH, United States; ^4^Department of Molecular, Cellular, and Biomedical Sciences, University of New Hampshire, Durham, NH, United States; ^5^Department of Biological Sciences, University at Albany, Albany, NY, United States; ^6^Department of Biomedical and Nutritional Sciences, University of Massachusetts Lowell, Lowell, MA, United States; ^7^Department of Molecular, Cellular, and Biomedical Sciences, Hubbard Center for Genome Studies, University of New Hampshire, Durham, NH, United States

**Keywords:** type 2 diabetes, gastrointestinal microbiome, inflammation, metabolic endotoxemia, Bhutanese refugee adults

## Abstract

**Introduction:**

South Asian refugees experience a high risk of obesity and diabetes yet are often underrepresented in studies on chronic diseases and their risk factors. The gut microbiota and gut permeability, as assessed through circulating lipopolysaccharide binding protein (LBP), may underlie the link between chronic inflammation and type 2 diabetes (T2D). The composition of the gut microbiota varies according to multiple factors including demographics, migration, and dietary patterns, particularly fiber intake. However, there is no evidence on the composition of the gut microbiota and its relationship with metabolic health in refugee populations, including those migrating to the United States from Bhutan. The objective of this study was to examine glycemic status in relation to LBP, systemic inflammation fiber intake, and gut microbiota composition in Bhutanese refugee adults residing in New Hampshire (*n* = 50).

**Methods:**

This cross-sectional study included a convenience sample of Bhutanese refugee adults (*N* = 50) in NH. Established bioinformatics pipelines for metagenomic analysis were used to determine relative genus abundance, species richness, and alpha diversity measures from shallow shotgun sequences. The relationships between inflammatory markers, gut microbiota composition, dietary fiber, and glycemic status were analyzed.

**Results:**

We identified a substantial chronic disease burden in this study population, and observed a correlation between glycemic status, LBP, and inflammation, and a correlation between glycemic status and gut microbiome alpha diversity. Further, we identified a significant correlation between proinflammatory taxa and inflammatory cytokines. SCFA-producing taxa were found to be inversely correlated with age.

**Conclusion:**

To date, this is the most comprehensive examination of metabolic health and the gut microbiome in a Bhutanese refugee population in NH. The findings highlight areas for future investigations of inflammation and glycemic impairment, in addition to informing potential interventions targeting this vulnerable population.

## Introduction

The gut microbiota has been identified as a key mediator of cardiometabolic health with implications in inflammation and glycemic control. Moreover, alterations in human gut microbiota composition, known as dysbiosis, are associated with increased risk for type 2 diabetes (T2D) (1). Gut dysbiosis often leads to intestinal hyperpermeability and increased plasma levels of lipopolysaccharide (LPS), defined as metabolic endotoxemia (2, 3). This pathway is likely responsible for low-grade inflammation exhibited in T2D (4), which is characterized by increased secretion of pro-inflammatory cytokines, including tumor necrosis factor alpha (TNF-α) and interleukin (IL) 6, and acute phase proteins such as C-reactive protein (CRP) levels into systemic circulation (2). Fiber intake has been highlighted as a key modulator of gut microbiota composition and its production of metabolites, which can impact intestinal permeability, inflammation, and glycemic control (5). However, more evidence is necessary to fully elucidate specific microbe-host interactions that contribute to the pathology of metabolic diseases and potential modifiable risk factors, specifically in human models.

Microbial composition is population-specific and varies drastically across ethnic groups, demographics, lifestyles (6), environmental factors, and migration (7, 8). Chronic disease risk tends to be higher for refugee populations in the US compared to non-Hispanic White (NHW) populations (9). The Bhutanese refugee population in NH not only faces higher rates and risk of T2D (10–12) but is underrepresented in current research on the gut microbiome. Moreover, South Asian adults, including those from Bhutan and Nepal, experience a higher prevalence of prediabetes and T2D as compared to non-Hispanic White, African American, and Hispanic/Latino adults (13). The objectives of this article were to quantify the cross-sectional associations between glycemic status and inflammatory markers (IL-6, TNF-α, and LBP), fiber intake, and the microbiome (richness, diversity, and composition) among Bhutanese refugee adults. We hypothesized that T2D and poor glycemic control are characterized by higher inflammation and LBP concentrations, lower microbial richness, and lower fiber intake in this population. This work addresses the significant burden of metabolic diseases among vulnerable populations and shows unprecedented results on the associations between the gut microbiota, inflammation, and glycemic status in this refugee population.

## Materials and methods

### Study population

**FIGURE 1:**
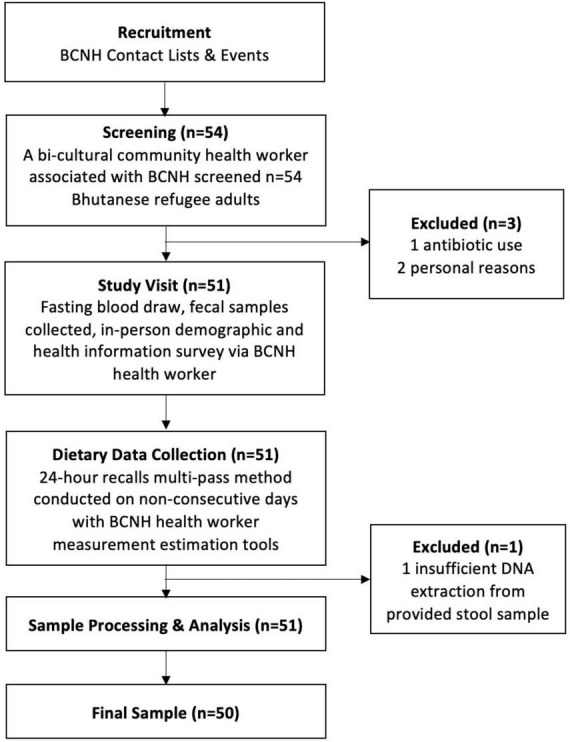
Participant recruitment and data collection in coordination with Building Community in New Hampshire (BCNH).

Participants were recruited in collaboration with a non-profit community-based organization, Building Community in New Hampshire (BCNH), in the areas of Manchester and Concord, New Hampshire (Figure 1). Fifty-four individuals from a convenience sample of culturally insulated adult Bhutanese refugees were recruited as part of a previously completed study led by one of the co-authors (Bigornia). The original study criteria included Bhutanese refugee adults that were 18 years or older and resource limited. The latter was determined by eligibility to receive SNAP (Supplemental Nutrition Assistance Program) benefits. Participants were excluded if they indicated moving within 2 months, being pregnant or trying to become pregnant, or prescribed antibiotics within the past 6 months. After screening, 50 participants were included in this cross-sectional study. All the participants signed informed consent. Recruitment, consent, and data collection were conducted by a trained community health worker who identified as Bhutanese refugee and spoke Nepali fluently. The research protocol was approved by the University of New Hampshire Institutional Review Board (IRB #8042).

### Dietary intake

Three supervised 24-h recalls were conducted on non-consecutive days by a bilingual and bicultural community health worker trained to collect dietary information. Participants were instructed in person on how to report their dietary intake and estimate portion sizes. The community health worker directly entered 24-h recall data into Nutrition Data System for Research (NDSR) (version 2019, Minneapolis, MN, USA). This software package and nutrient database was used to estimate nutrient intakes based on 24-h recall food weights. Fiber consumption as well as other nutrients and foods were averaged across the three 24-h recall days.

### Blood sampling, processing, and biomarker assessment

After a 12-h fast, a sample of approximately 35 ml of blood was collected by a trained phlebotomist, using EDTA (14 ml total), lithium heparin (10 ml), and serum (10 ml) vacutainers. All vacutainer samples were transported on ice to the University of New Hampshire (UNH) and centrifuged at 3,000 RPM for 10 min. Post centrifugation, samples were aliquoted into 0.5 ml portions into six 2 ml cryotubes and frozen at −80^°^C. TNF-α, IL-6, and insulin were measured *via* enzyme-linked immunosorbent assays (ELISA). High-sensitivity CRP was calculated using a clinical chemistry analyzer. Glycosylated hemoglobin (HbA1c) was measured from whole blood samples using a Siemens DCA Vantage analyzer. Fasting glucose was measured using a clinical chemistry analyzer. Homeostasis model assessment-estimated insulin resistance (HOMA-IR), a measure of insulin resistance, was calculated by fasting serum insulin (μIU/ml) × fasting plasma glucose (mg/dL)/405, with a higher value indicating a higher degree of insulin resistance. Participants were classified as having T2D if they met any of the following criteria: self-reported diabetes, HbA1c of 6.5% or higher, or use of a diabetes medication. Prediabetes was defined as 6.5% ≥ HbA1c ≥ 5.7% (14). LBP was measured using an electrochemiluminescence technology (MesoScale Discovery). Briefly, carbon electrodes of the assay plates bind biological reagents attaching biomarkers from participant serum samples. Biomarker concentrations are measured as light intensity emitted from electrochemiluminescence labels conjugated with detection antibodies as electricity is applied to the assay plate. This method allows for high sensitivity, broad dynamic range, and reduced time compared to the traditional ELISA method.

### Fecal sampling and processing

Fecal samples were collected in DNA preservation solution and transported at room temperature. DNA from fecal samples was extracted using Zymo Research Quick-DNA Fecal/Soil Microbe kits. Extracted DNA was sequenced by the Hubbard Center for Genome Studies (University of New Hampshire) using shallow shotgun metagenomic sequencing (Illumina NovaSeq) (15).

Raw sequences were processed using the Metagenome-atlas snakemake workflow (16). The workflow conducted quality control processes using the *BBtools* package. PCR duplicates were removed (clumpify). Adapters were removed and reads were trimmed and filtered (BBduk). Host contamination was removed (BBsplit) based on a masked HG19 reference. Quality controlled sequence files (fastq files) were used in subsequent components of the atlas workflow and in further pipelines. *Sourmash gather* (scaled 1000) was used to generate FracMinHash sketch from the samples and the GTDB rs207 full reference database (17). *Sourmash taxonomy* provided taxonomic lineage information for *Sourmash gather* results (18). Results were then converted into a phyloseq object for further analyses and processing using the pipeline by Callahan et al. (19). Rarefying or proportions were not used to normalize data for sequencing depth (20). All richness and diversity measures were calculated using the raw, unfiltered taxa data. The observed species richness was calculated by counting the number of unique species in each sample. Only phyla prevalent in 5% or more samples were kept in filtered count data. To calculate the relative abundance of a particular genus and species, the metagenomic species table was further filtered to only include species with a mean greater than 10^–5^ and two data frames were agglomerated, one to the species and one to the genus levels. Counts were converted to relative abundance and normalized to the median sequencing depth. Relevant taxonomic groups were identified, and the relative abundance of given groups was extracted. The species *Clostridium coccoides*, *Ruminococcus productus*, *Clostridium cocleatum*, *Bifidobacterium catenulatum*, *Eubacterium hallii*, and *Akkermansia municiphila* were excluded as they were not present in the filtered dataset.

### Other variables

Demographics and additional health information were collected *via* an in-person survey. Participants self-reported having a history of heart attack, heart disease (other than heart attack), and or stroke. Those who reported experiencing any of these health conditions were categorized as having cardiovascular disease (CVD). Validated methods were followed for calculating physical activity (PA) score and food security score (FSS) (21). PA scores are interpreted as low (<30), moderate (30–39), or high (40+) (21). FSS was dichotomized into <3 food secure and ≥3 food insecure. Anthropometrics, height and weight, were obtained during the study visit, and body mass index (BMI) was calculated as kg/m^2^. Overweight was defined as a BMI > 23 kg/m^2^ and obesity as a BMI ≥ 25 kg/m^2^ (22). Medication use, smoking status, years in the US, household size, and high school completion were all obtained *via* in-person survey. Differences in age, sex, cardiovascular disease (CVD), BMI, smoking status, medication use, years in the US, PA score, household size, FSS, and high school completion were compared according to T2D status. Age and sex were used as covariates in all analyses.

### Statistical analysis

Analyses were conducted using SAS 9.4 (Cary, NC) and R. Between-group differences were assessed using parametric (ANCOVA, *t*-test) and non-parametric (Wilcoxon, Fishers Exact) analyses depending on data normality. HOMA-IR and HbA1c followed non-normal distributions and were log transformed for linear regression analyses. Spearman correlations were used to examine continuous variable associations. The Benjamin Hochberg method was used to adjust for multiple testing in the microbiome analysis. All models included the covariates age and gender. The rationale for this is that the adult gut is relatively stable until the process of aging and disruption of homeostatic control diminishes the stability. Older age is accompanied by increased proinflammatory status and decreasing adaptive immunity. Aging can influence the gut microbiota through its various impacts of gut function including gastric motility, hypochlorhydria, and changes in the enteric nervous system (23). Further, risk factors of chronic disease and microbiota composition vary by sex (24). Additionally, fiber consumption levels often vary between sexes (25). Further, linear regression was used to assess predictors of diabetes markers and logistic regression was used to predict T2D, with age and sex as covariates (Table 1).

**TABLE 1:** Regression models predicting T2D and glycemic impairment.

Logistic regression models	
Model 1	T2D = CRP + IL6 + TNF-α + LBP + Age + Sex
Model 2	T2D = Total fiber + Insoluble fiber + Soluble fiber + Whole grains + Age + Sex
Model 3	T2D = Observed species richness + Age + Sex
**Linear regression models**	
Model 4	Log transformed HOMAIR = LBP + Observed richness + Age + Sex
Model 5	Log transformed HbA1c = LBP + Observed richness + Age + Sex

Alpha diversity was compared according to T2D status using the Wilcoxon rank-sum test. Spearman correlation analysis was used to quantify the relationship between observed species richness and other markers of glycemic status and inflammation. The relative abundance of fecal pro-and anti-inflammatory bacteria and SCFA-producing bacteria, identified through a literature review, was compared by glycemic status. Additionally, the relative abundance of fecal pro-and anti-inflammatory bacteria and SCFA-producing bacteria was correlated to multiple biomarkers and demographic characteristics using a spearman correlation matrix. All correlations were subsequently corrected using the Benjamini-Hochberg method.

## Results

### Population characteristics and health behaviors

The median (interquartile range, IQR) age of the 50 participants (82% female) was 49.5 (24.0) years (Table 2). Forty-two percent of the participants were classified as having T2D and 10% as having cardiovascular disease (CVD), excluding hypertension. The prevalence of prediabetes was 34% in the non-T2D group, with individuals having a median (IQR) HbA1c of 5.6 (0.5)%. The median BMI was 27 (5.4) kg/m^2^ and 92% of participants were categorized as being overweight or having obesity. Eight percent of participants reported habitual smoking. At the time of data collection, participants had spent a median of 8 (4) years in the US and 14% completed high school. The median physical activity score was calculated as 27 (3.3) (i.e., moderate, on average). Although all participants were SNAP-eligible, 90% of them reported a food security score of <3, which corresponds to food security (21).

**TABLE 2:** Demographics by type 2 diabetes status.

	All participants					No-T2D					T2D					
	n	Median or %	Min	Max	IQR	n	Median or %	Min	Max	IQR	n	Median or %	Min	Max	IQR	*P*-value
**Clinical**																
CVD (% with CVD)^†^	5	10.0				1	3.5				4	19.1				0.148^†††^
BMI (kg/m^2^)	50	27.1	20.0	38.9	5.4	29	27.1	20.0	38.9	4.4	21	27.0	20.1	35.9	7.7	0.891^††^
BMI category (% overweight/obese)	46	92.0				26	90.0				20	95.2				0.630^†††^
Smoking status (% smoker)	4	8.0				1	3.5				3	14.3				0.297^†††^
Physical activity score	49	27.0	24.2	38.3	3.3	28	27.3	25.2	37.6	2.8	21	26.8	24.2	38.3	3.3	0.620^††^
Metformin (% use)	14	28.0				0	0.0				14	66.7				<0.0001*
Statin (% use)	15	30.0				4	13.8				11	52.4				0.003*
NSAID (% use)	4	8.0				1	3.5				3	14.3				0.297^†††^
PPI (% use)	13	26.0				5	17.2				8	38.1				0.097
**Demographic**																
Age (y)	50	49.5	18.0	72.0	24.0	29	45.0	18.0	71.0	22.0	21	58.0	31.0	72.0	21.0	0.014*^††^
Sex (% female)	41	82.0				25	86.2				16	76.2				0.464^†††^
Years in US (y)	49	8.0	2.0	28.0	4.0	28	8.0	4.0	28.0	4.0	21	8.0	2.0	12.0	6.0	0.654^††^
Household size	50	4	1	8	2	29	4	1	7	2	21	4	1	8	2	0.279^††^
Food security (% food secure)	45	90.0				26	89.7				19	90.5				1.000^†††^
High school completion (% completed)	7	14.0				4	13.8				3	14.3				1.000^†††^
**Dietary intake**																
Total dietary fiber (g)	50	20.3	6.2	40.5	11.23	29	18.9	6.2	36.4	8.3	21	20.9	13.1	40.5	10.8	0.065^†††^
Insoluble fiber (g)	50	16.0	3.9	34.0	9.4	29	15.0	3.9	30.3	7.6	21	18.3	9.7	34.0	6.7	0.062^††^
Soluble fiber (g)	50	3.9	1.4	9.4	1.8	29	3.6	1.4	5.6	1.32	21	4.1	2.0	9.4	1.8	0.035*^††^
Whole grains (oz equivalents)	50	0.6	0.0	4.0	1.1	29	0.4	0.0	3.0	0.8	21	1.1	0.0	4.0	1.5	0.028*^††^
Daily caloric intake (kcal)	50	1245	586	2535	382	29	1207	586	2535	417	21	1251	696	2193	185	0.709^††^

*Statistically significant at alpha level 0.05.

^†^CVD excluding hypertension.

^††^Wilcoxon used to generate p-value.

^†††^Fisher’s exact test used; assumptions were not met for chi-squared.

The prevalence of overweight and obesity for participants with T2D was 90 and 95.2% for participants without T2D, but the median BMI was not significantly different between both groups. The median age of the T2D group, 58 (21) years, was notably higher than the non-T2D group, 45 (22) years (*p* = 0.014) (Table 2). Both groups showed comparable years lived in the US and physical activity level. Fiber intake and whole grain consumption were significantly higher in the T2D group (*p* = 0.035 and *p* = 0.028, respectively). No misreporters were identified upon application of the Mifflin equation or cutoffs for implausible dietary intake (data not shown) (26).

### Glycemic status and its correlation with inflammation and LBP

Although the T2D group had significantly higher median HbA1c and glucose compared to the non-T2D (both *p* < 0.001), 34.5% of participants without T2D qualified as having prediabetes (Table 3). Among all participants, 28, 30, 8, and 26% reported taking metformin, statins, non-steroidal anti-inflammatory drugs (NSAIDs), or proton pump inhibitors (PPIs), respectively. Metformin and statin use were higher in the T2D group, 66.7 and 52.4%, respectively, as compared to the non-T2D group 0 and 13.8%, respectively (Table 2).

**TABLE 3:** Clinical factors, dietary intake, and inflammatory markers.

	All participants					Non-T2D					T2D					
	n	Median	Min	Max	IQR	n	Median	Min	Max	IQR	n	Median	Min	Max	IQR	*P*-value
**Glycemic status markers**																
HOMA-IR	50	4.3	0.2	28.3	5.7	29	3.8	0.2	27.1	5.6	21	6.6	1.2	28.3	5.4	0.195
HbA1c (%)	50	5.9	4.9	10.4	1.2	29	5.6	4.9	6.1	0.5	21	6.9	5.6	10.4	1.5	<0.001*
FPG (mg/dL)	50	120.0	88.0	274.0	36.0	29	108.0	88.0	141.0	17.0	21	155.0	97.0	274.0	49.0	<0.001*
Insulin (μIU/ml)	50	14.4	0.9	81.4	14.9	29	15.2	0.9	81.4	14.9	21	13.4	5.1	58.8	13.9	1.00
**Inflammatory markers**																
CRP (mg/L)	50	2.8	0.1	29.0	3.8	29	2.4	0.1	29.0	3.6	21	3.2	0.5	18.6	4.5	0.568
IL6 (pg/ml)	49	2.0	0.0	6.9	2.6	29	2.0	0.0	6.8	3.2	20	1.9	0.0	6.9	2.5	0.745
TNFα (pg/ml)	49	8.1	0.0	51.1	5.6	29	7.7	0.0	51.1	8.2	20	8.3	0.0	19.7	5.3	0.919
LBP (μg/ml)	50	4.2	1.3	8.4	2.3	29	4.0	1.5	5.9	2.3	21	4.6	1.3	8.4	1.6	0.398
Leptin (pg/ml)	50	8638.9	360.9	93520.6	13691.3	29	8700.2	360.9	93520.6	12802.6	21	7232.6	514.2	67876.1	17672.1	0.479
**Lipid profile**																
Total cholesterol (mg/dL)	50	179.5	87.0	281.0	57.0	29	190.0	129.0	281.0	39.0	21	162.0	87.0	247.0	71.0	0.053
LDL cholesterol (mg/dL)	50	80.0	28.0	143.0	41.0	29	90.0	47.0	143.0	26.0	21	65.0	28.0	137.0	41.0	0.028*
HDL cholesterol (mg/dL)	50	41.5	22.0	64.0	13.0	29	43.0	31.0	55.0	10.0	21	39.0	22.0	64.0	15.0	0.154
Triglycerides (mg/dL)	50	134.0	35.0	454.0	112.0	29	113.0	35.0	454.0	72.0	21	158.0	65.0	338.0	114.0	0.004*

*Statistically significant at alpha level 0.05.

Lipids, excluding triglycerides, were lower in the T2D group, potentially as a result of pharmacological therapy. Even though there were no differences in inflammatory cytokines, CRP, or LBP between the T2D and non-T2D groups (Table 3), CRP had a weak positive correlation with HbA1c (Rho = 0.39, *p* = 0.006) and FPG (Rho = 0.31, *p* = 0.033) (Table 4). LBP was also moderately correlated with HbA1c (Rho = 0.42, *p* = 0.003) and FPG (Rho = 0.42, *p* = 0.003), and only weakly correlated to HOMA-IR (Rho = 0.35, *p* = 0.016) (Table 4). Inflammatory cytokines and dietary intake measures were not associated with glycemic status (Table 4).

**TABLE 4:** Partial spearman correlation analysis of diabetes markers, inflammatory markers, and dietary intake.

	HOMA-IR			HbA1c			FPG			Insulin		
	n	Rho	*P*-value	n	Rho	*P*-value	n	Rho	*P*-value	n	Rho	*P*-value
**Inflammatory markers**												
C-reactive protein (mg/L)	50	0.23	0.111	50	0.39	0.006*	50	0.31	0.033*	50	0.18	0.230
Interleukin-6 (pg/ml)	49	0.22	0.132	49	-0.02	0.883	49	0.19	0.206	49	0.21	0.166
Tumor necrosis factor alpha (pg/ml)	49	0.22	0.142	49	0.16	0.294	49	0.13	0.375	49	0.17	0.266
Liposaccharide-binding protein (μg/ml)	50	0.35	0.016*	50	0.42	0.003*	50	0.42	0.003*	50	0.24	0.105
Leptin (pg/ml)	50	0.27	0.068	50	0.12	0.398	50	0.07	0.645	50	0.27	0.063
**Dietary intake**												
Total dietary fiber (g)	50	0.06	0.708	50	-0.03	0.847	50	0.01	0.943	50	0.04	0.806
Insoluble fiber (g)	50	0.04	0.798	50	-0.04	0.798	50	0.00	0.978	50	0.01	0.934
Soluble fiber (g)	50	0.08	0.587	50	0.02	0.882	50	0.01	0.971	50	0.08	0.611
Whole grains (oz equivalents)	50	0.12	0.427	50	0.14	0.347	50	-0.06	0.672	50	0.12	0.406
Daily caloric intake (kcal)	50	-0.06	0.664	50	-0.06	0.678	50	-0.04	0.781	50	-0.08	0.590

Partial spearman correlation adjusted for age and sex.

*Statistically significant at alpha level 0.05.

Regression models used to assess predictors of T2D are shown in Table 5. Age was the only significant predictor of T2D in any of the logistic regression models (Table 5). A one unit increase in LBP was associated with a 27% increase in HOMA-IR (*p* = 0.004) in model 4 and a 4% increase in HbA1c (*p* = 0.010) in model 5 (Table 6). Both models included observed species richness, age, and sex as covariates.

**TABLE 5:** Logistic regression models 1–3 predicting T2D status.

Model	Variable	Coefficient	Odds ratio	95% CI	*P*-value
Model 1	CRP	0.00	1.00	(0.86, 1.13)	0.985
	LBP	-0.17	0.84	(0.52, 1.34)	0.462
	Age	-0.06	0.95	(0.90, 0.99)	0.019*
	Sex	0.44	1.55	(0.33, 7.36)	0.585
Model 2	Soluble fiber	-0.30	0.74	(0.46, 1.20)	0.226
	Whole grains	-0.45	0.64	(0.29, 1.42)	0.272
	Age	-0.04	0.96	(0.92, 1.01)	0.104
	Sex	0.23	1.26	(0.24, 6.69)	0.785
Model 3	Observed species richness	0.01	1.01	(1.00, 1.02)	0.174
	Age	-0.05	0.95	(0.91, 1.00)	0.022*
	Sex	0.39	1.48	(0.32, 6.92)	0.619

*Statistically significant at alpha level 0.05.

**TABLE 6:** Linear regression models 4–5 predicting T2D status.

	Variable	Coefficient	SE	*P*-value
Model 4 HOMAIR	LBP	1.27	0.08	0.004*
	Observed species richness	1.00	0.00	0.587
	Age	1.00	0.01	0.620
	Sex	1.13	0.30	0.691
Model 5 HbA1c	LBP	1.04	0.02	0.010*
	Observed species richness	1.00	0.00	0.795
	Age	1.00	0.00	0.144
	Sex	0.99	0.06	0.875

*Statistically significant at alpha level 0.05.

### Association between gut microbiota composition and glycemic status

**FIGURE 2:**
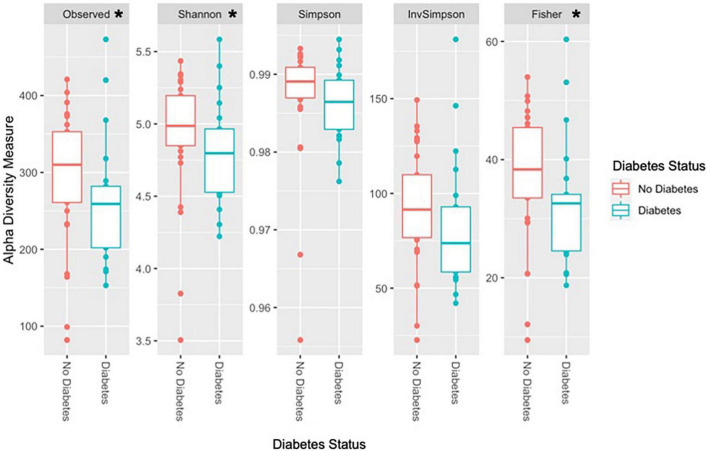
Diversity measures according to T2D status. *Statistically significant at alpha level 0.05. Observed species richness, Shannon, and Fisher diversity were significantly higher in the non-T2D group compared to the T2D group. Other alpha diversity measures, Simpson and inverse Simpson were higher in the non-T2D group but did not reach statistical significance.

Observed species richness and alpha diversity measures were significantly higher in the non-T2D group than in the T2D group (Figure 2). Specifically, observed species richness, Shannon, and Fisher diversity measures were statistically significant (Table 7).

**TABLE 7:** Wilcoxon rank sum test of alpha diversity measures by T2D status.

	W	*P*-value
Observed species richness	407	0.045*
Shannon	405	0.049*
Simpson	400	0.061
Inverse Simpson	400	0.061
Fisher	409	0.040*

*Statistically significant at alpha level 0.05.

**FIGURE 3:**
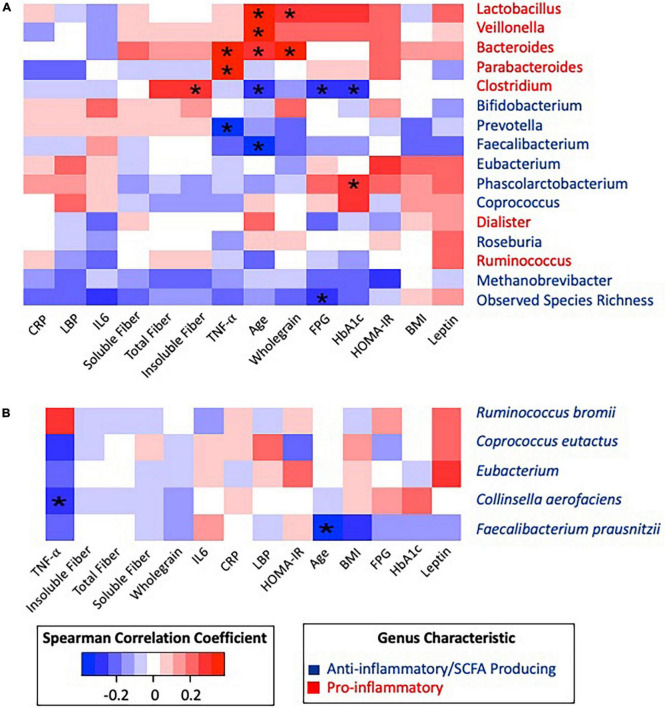
Spearman correlation matrix heatmap of inflammatory associated taxonomic groups with clinical biomarkers and dietary data. **(A)** Spearman correlation matrix of inflammatory associated genera and observed species richness with clinical biomarkers and dietary data. **(B)** Spearman correlation matrix of inflammatory associated species with clinical biomarkers and dietary data. *Statistically significant at alpha 0.05. *P*-values were adjusted using the Benjamini–Hochberg method. All significance was lost after BH correction.

The most abundant phyla were *Firmicutes*, *Bacteroidetes*, and *Actinobacteria*. No significant differences in relative abundance of proinflammatory or SCFA-producing species were identified according to T2D status. Age was positively correlated with *Lactobacillus* (Rho = 0.333, *p* = 0.018), *Veillonella* (Rho = 0.325, *p* = 0.021), and *Bacteroides* (Rho = 0.306, *p* = 0.031) and negatively correlated with *Clostridium* (Rho = −0.292, *p* = 0.040), *Faecalibacterium* (Rho = −0.386, *p* = 0.006) (Figure 3A), and the species *Faecalibacterium prausnitzii* (Rho = −0.374, *p* = 0.007) (Figure 3B). Given the differences in fiber intake, microbiome composition, and glycemic status according to age, a partial correlation analysis adjusting for age was conducted (Supplementary Figure 1).

Pro-inflammatory genera *Parabacteroides* (Rho = 0.339, *p* = 0.017) and *Bacteroides* (Rho = 0.363, *p* = 0.010) were positively correlated with the inflammatory cytokine TNF-α, while anti-inflammatory *Prevotella* was negatively correlated (Rho = −0.320, *p* = 0.010) with TNF-α. After adjusting for age, all associations with TNF-α remained significant. Observed species richness was negatively correlated with FPG (Rho = −0.280, *p* = 0.049), and HbA1c was positively correlated with *Phascolarctobacterium* (Rho = 0.293, *p* = 0.039). These correlations between glycemic status and taxonomic groups remained significant after adjusting for age.

When considering dietary fiber, positive correlations were identified between *Lactobacillus* and whole grains (Rho = 0.307, *p* = 0.030), Bacteroides and whole grains (Rho = 0.364, *p* = 0.009), and *Clostridium* and insoluble fiber (Rho = 0.295, *p* = 0.037). Furthermore, *Clostridium* was negatively correlated to FPG (Rho = −0.293, *p* = 0.039) and HbA1c (Rho = −0.300, *p* = 0.035). However, after adjusting for age, *Clostridium* became positively correlated to insoluble fiber (Rho = 0.356, *p* = 0.013) and total fiber (Rho = 0.323, *p* = 0.025).

## Discussion

This work shows the characterization of glycemic status in relation to clinical risk factors, with an emphasis on inflammation and metabolic endotoxemia, dietary intake, and gut microbiota composition which, to our knowledge, is unprecedented in Bhutanese refugee adults in the US. The chronic disease burden of this study population was substantial, with particularly high rates of overweight/obesity, prediabetes, and diabetes. Glycemic impairment (e.g., higher HOMA-IR and HbA1c) was characterized by higher levels of CRP and LBP, while diet and inflammatory cytokines were unrelated to glycemic markers. Observed species richness, Shannon, and Fisher alpha diversity were higher in the non-T2D group than in the T2D group.

There is a strong impetus to better characterize the gut microbiota in relation to health outcomes in vulnerable populations that are underrepresented in research. Many Nepalese-speaking Bhutanese individuals have resettled in the United States due to political, social, and economic restrictions in the 1990s and a lack of successful integration in Nepal (27). This population qualified as living in an isolated social enclave. Eighty-six percent of the convenience sample had not completed high school and all participants qualified for SNAP benefits based on household income. This situation has placed this population, and other similarly vulnerable populations in the US, at a particularly higher risk for malnutrition and chronic disease.

Limited studies exist on obesity and related chronic health conditions in Bhutanese refugee adults in the United States. Previous studies focused primarily on infectious diseases and dietary deficiencies (28). This study identified a substantial chronic disease burden among this convenience sample of Bhutanese refugees, with an alarming prevalence of overweight/obesity comparable to the US national average (29). To compound the comparable weight status measures, South Asian populations have a higher risk of cardiometabolic diseases at lower BMIs than other ethnic groups (28). Furthermore, the prevalence of overweight/obesity in this study (92%) was higher than other studies in Bhutanese refugee communities in the US (11). Given the high prevalence of obesity, greater attention to culturally relevant, economically feasible interventions and education are warranted in the Bhutanese refugee community, specifically for those who are among SNAP eligible groups. Evidence emphasizes the need for relevant lifestyle and dietary change education programs and interventions to address the overweight/obesity prevalence among this vulnerable population (28).

The proportion of diabetes was higher among the study participants (42%) than the general US population (8.2%), and surpassed prevalence measures in other US Bhutanese refugee communities (6–14%) (9, 30). Additionally, a high prevalence of prediabetes was identified in the non-T2D group. South Asian populations are at a higher risk of insulin resistance and T2D than non-Hispanic white populations, yet to our knowledge, this is the first study to have explored inflammatory markers in relation to diabetes in the Bhutanese refugee population (22). Consistent with previous findings, CRP, an indicator of systemic inflammation, was correlated with diabetes markers (31). Additionally, LBP was associated with HbA1c and FPG and weakly with HOMA-IR. LBP was also found predictive of HOMA-IR and HbA1c, replicating trends in previous studies exploring LBP and diabetes (4). It is unclear whether LBP increases as a result of endotoxemia or systemic inflammation present in chronic diseases, but it is often used as a proxy for endotoxemia or impaired gut barrier integrity (4). Findings suggest a potential connection between gut function, inflammation, and glycemic markers. The variation in LBP concentrations in our study was small, which could be attributed to an insufficient number of metabolically healthy participants and high proportion of prediabetes in the non-T2D group. Studies with a greater range of metabolic profiles may find stronger correlations between inflammation indicators and diabetes markers.

Inflammatory cytokine concentrations were unexpectedly not associated with diabetes markers. Given that obesity is associated with low-grade inflammation, characterized by elevated inflammatory cytokines, which in turn may induce systemic insulin resistance (32), a potential explanation for these findings may be that the high proportion of overweight and obesity in both the non-T2D group and T2D group, and the high prevalence of pre-diabetes in the non-T2D group (33, 34) may have attenuated expected differences in cytokines according to glycemic status.

Dietary intake, including fiber and whole grain consumption, was not associated with glycemic impairment. Specific dietary components, including red meat consumption, have been shown to influence chronic disease in Bhutanese refugee adults in the US (28). Increasing fiber intake has been suggested to reduce the risk of T2D; however, this relationship has not been studied extensively within the US Bhutanese refugee population (35). Median fiber intake and whole grain consumption were higher in the T2D group (*p* = 0.0354 and *p* = 0.0279, respectively). However, higher fiber intake may be due to reverse causality of a T2D diagnosis, which may translate into high motivation for nutrition and health education, providing a pivotal opportunity for nutrition interventions (27).

Studies suggest that at least 5 years after resettlement and subsequent acculturation precede higher risk of chronic disease (36). The median time spent in the US for the study population was 8 (4) years, providing sufficient time to see the impact of US acculturation among this population. However, previous studies, such one including Ohio Bhutanese refugee women, have failed to find associations between chronic diseases and length of time in the US (28). Our findings indicate no correlation between years in the US and T2D prevalence. Kumar et al. suggest that chronic disease risk factors may develop at refugee camps and associated lifestyle changes before relocation to the US (11). Irrespective of the origin of such chronic diseases, interventions are needed to address the significant proportion of Bhutanese refugee adults succumbing to risk factors of chronic disease and those already suffering from various chronic diseases.

Utilizing the strengths of shallow shotgun metagenomic sequencing, this study provided a thorough characterization of the gut microbiota composition and its capabilities in relation to T2D and inflammation. The T2D group was expectedly characterized by lower average observed species richness and alpha diversity measures. Although inconsistently observed, low microbial richness has been associated with obesity, insulin resistance, and low-grade inflammation (37). Low richness and diversity have been mostly identified as markers of gut dysbiosis. However, we did not observe an association between richness, inflammation, or LBP (2, 3). The underlying explanation for these unexpected results could be also attributed to the high prevalence of overweight, obesity, and glycemic impairment in this population.

Additional outcomes were aligned with the hypothesis that inflammation and glycemic impairment are correlated with gut microbiome composition in this population. *Bacteroides* has been suggested to dominate gut profiles in chronic disease and inflammatory states, which is consistent with our observed correlation between the genera *Parabacteroides* and *Bacteroides* and TNF-α (37).

Age appeared to be the strongest characteristic associated with gut microbiota composition in this population. This study observed negative correlations with SCFA-producing genera and species (*Faecalibacterium* and *Faecalibacterium prausnitzii*). SCFA-producing potential typically decreases as individuals age and the gut composition alters over time (38). Additionally, *Bacteroides* and *Lactobacillus* were positively correlated with age (38). However, in this study, the T2D was significantly older than the non-T2D group. Given that over a third of the participants in the non-T2D group have prediabetes, age may mask differences in composition attributed to the disease progression of T2D.

While culturally competent health treatments are often inaccessible to Bhutanese refugee communities in the US (27), Krause found that yoga and mindfulness activities were perceived as medicinal and therapeutic by US Bhutanese refugee adults (27). Stress and physiological states have been shown to influence the gut microbiota bidirectionally, suggesting a potential role for mindfulness interventions to influence this bidirectional relationship (39). Exercise broadly has also been well-documented to influence the gut microbiota (39). Bhutanese refugee populations could benefit from these types of interventions, which would also support the cultural identity and social support of these communities (27, 39). Further, studies highlight a need for culturally appropriate and feasible dietary interventions that recognize the importance of dietary practices as part of cultural identity (28). An emphasis on the mind–body connection is essential in supporting the ideas and values that are the foundation of the Bhutanese refugee community (27). Thus, culturally appropriate interventions that emphasize mind–body connections should be explored in this and other underrepresented populations, particularly ones that value the synergies among the body, mind, and health.

### Strengths and limitations

To date, this study has been the most comprehensive examination of fiber intake, inflammation, and glycemic status in Bhutanese refugee adults in NH. Three 24-h recalls, the gold standard for dietary assessment, were utilized to assess fiber intake, reducing the chance of misclassification errors. Additionally, participant facing interactions and data collection were conducted by a bilingual and bicultural community health.

Despite the strengths of our study, several limitations should be noted. Utilizing a convenience sample was necessary for the feasibility of this study, however, it may limit the generalizability of results. Further, the relatively small sample size of *n* = 50, could have contributed to type 2 error. A larger and more representative sample is recommended for future studies on the Bhutanese refugee population. Another limitation to this study was the inability to account for medication use in our statistical analysis, which may explain some unexpected findings and potentially confounded the analysis of the microbiome. Medication use was not an exclusionary factor in this study thus many participants were on a variety of medications targeting their chronic diseases or risk factors, including Metformin, Statins, NSAIDs, and PPIs. Unexpected results in lipid profiles are likely attributable to medication use, as a large proportion of the participants with T2D were taking statins, which lower lipid levels (40). In addition to clinical biomarkers, medication use may have also confounded gut microbiome composition and function. Metformin, Statins, NSAIDs, and PPIs are known to potentially influence the gut microbiome and glycemic impairment (31, 40, 41). Accounting for medication use in the future is warranted to reduce any confounding and simplify interpretation of results. Shallow sequencing introduced additional bias in microbial analyses results through elimination of rare taxa and a focus on only the most abundant taxonomic groups (15). Utilization of deeper whole genome sequencing is recommended for future analyses of the gut microbiome composition and functional potential.

## Conclusion

To date, this is the most comprehensive examination of metabolic health, diet, and the gut microbiome in a Bhutanese refugee population in NH. Bhutanese refugee adults are at an increased risk of chronic diseases, such as T2D, and population-specific interventions are necessary to mediate the risk. Findings from this study highlight the need to investigate culturally relevant interventions to address chronic diseases and their risk factors. Future studies and interventions should focus on approaches to reduce chronic inflammation among this population with culturally tailored dietary and lifestyle changes.

## Data availability statement

The data presented in this study are deposited in the NCBI SRA repository, accession number: PRJNA912151.

## Ethics statement

The studies involving human participants were reviewed and approved by the University of New Hampshire Institutional Review Board for the Protection of Human Subjects in Research. The patients/participants provided their written informed consent to participate in this study.

## Author contributions

DM, BK, CL, TF, CA, CP, CO, MG, JS, WT, and SB: study design and implementation and data collection. BM, DM, SB, and MD: data analysis and interpretation of results. BM, SB, and MD: manuscript preparation. All authors approved the manuscript.

## Supplementary material

Supplementary Figure 1 Age-adjusted Spearman correlation matrix heatmap of inflammatory associated species with clinical biomarkers and dietary data stratified. **(A)** Age-adjusted spearman correlation matrix of inflammatory associated genera and observed species richness with clinical biomarkers and dietary data. **(B)** Age-adjusted Spearman correlation matrix of inflammatory associated species with clinical biomarkers and dietary data. *Statistically significant at alpha 0.05. *P*-values were adjusted using the Benjamini–Hochberg method. All significance was lost after BH correction.
