# Supplementary figures and images for: Association between inflammation, lipopolysaccharide binding protein, and gut microbiota composition in a New Hampshire Bhutanese refugee population with a high burden of type 2 diabetes
Source: Front Nutr. 2023 Jan 6;9:1059163. doi: 10.3389/fnut.2022.1059163 (PMC9852993; doi:10.3389/fnut.2022.1059163)

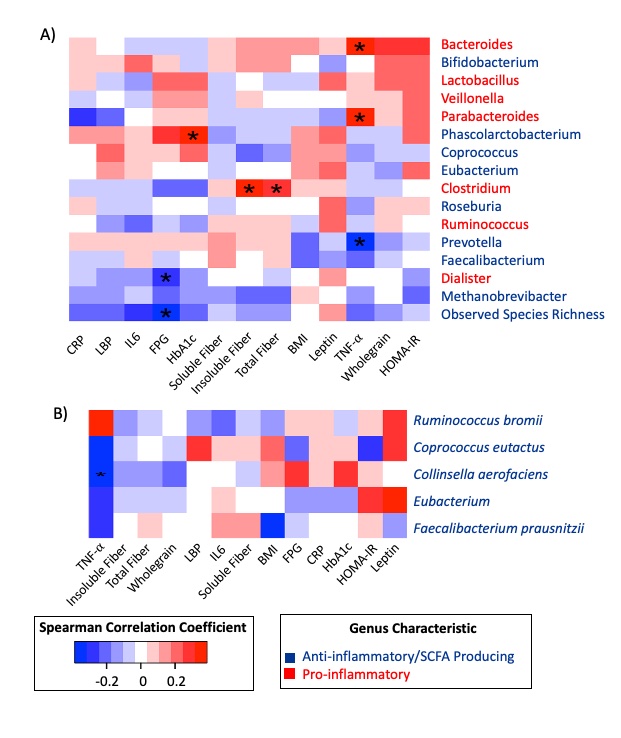

Supplement: Supplementary Figure 1 — Age-adjusted Spearman correlation matrix heatmap of inflammatory associated species with clinical biomarkers and dietary data stratified. (A) Age-adjusted spearman correlation matrix of inflammatory associated genera and observed species richness with clinical biomarkers and dietary data. (B) Age-adjusted Spearman correlation matrix of inflammatory associated species with clinical biomarkers and dietary data. *Statistically significant at alpha 0.05. P-values were adjusted using the Benjamini–Hochberg method. All significance was lost after BH correction. [file Image_1.JPEG]
